# Innate and Adaptive Immunity Alterations in Metabolic Associated Fatty Liver Disease and Its Implication in COVID-19 Severity

**DOI:** 10.3389/fimmu.2021.651728

**Published:** 2021-03-30

**Authors:** Patricia Lamadrid, Marta Alonso-Peña, David San Segundo, Mayte Arias-Loste, Javier Crespo, Marcos Lopez-Hoyos

**Affiliations:** ^1^ Transplant and Autoimmunity Group, Research Institute Marques de Valdecilla (IDIVAL), Santander, Spain; ^2^ Clinical and Translational Research in Digestive Pathology Group, Research Institute Marques de Valdecilla (IDIVAL), Santander, Spain; ^3^ Immunology Department, Marques de Valdecilla University Hospital, Santander, Spain; ^4^ Gastroenterology and Hepatology Department, Marques de Valdecilla University Hospital, Santander, Spain

**Keywords:** MAFLD, NASH, COVID-19, SARS-CoV-2, innate immunity, adaptive immunity

## Abstract

The coronavirus infectious disease 2019 (COVID-19) pandemic has hit the world, affecting health, medical care, economies and our society as a whole. Furthermore, COVID-19 pandemic joins the increasing prevalence of metabolic syndrome in western countries. Patients suffering from obesity, type II diabetes mellitus, cardiac involvement and metabolic associated fatty liver disease (MAFLD) have enhanced risk of suffering severe COVID-19 and mortality. Importantly, up to 25% of the population in western countries is susceptible of suffering from both MAFLD and COVID-19, while none approved treatment is currently available for any of them. Moreover, it is well known that exacerbated innate immune responses are key in the development of the most severe stages of MAFLD and COVID-19. In this review, we focus on the role of the immune system in the establishment and progression of MAFLD and discuss its potential implication in the development of severe COVID-19 in MAFLD patients. As a result, we hope to clarify their common pathology, but also uncover new potential therapeutic targets and prognostic biomarkers for further research.

## Introduction

The coronavirus infectious disease 2019 (COVID-19) pandemic caused by the severe acute respiratory syndrome coronavirus 2 (SARS-CoV-2) has emerged as a global challenge, affecting health and medical care but also economies and society. In an unprecedented way, researchers have unraveled its main clinical and epidemiological features, its pathogenesis and the mayor ways of transmission in record time. Knowledge and improved diagnostics and treatments are increasing day by day. It is not the aim of this review to summarize all these data, available elsewhere (1, 2), but we should highlight that SARS-CoV-2 infection is determined by the interaction of its spike protein with angiotensin converting enzyme 2 (ACE2) and further needs of transmembrane protease serine 2 (TMPRSS2) to initiate fusion and cell infection (3), although other receptors and proteases have been also involved (3–9). From here on, COVID-19 pathogenesis could evolve in several ways resulting in a wide range of symptoms and severity - from fully asymptomatic to death – (1). Host immune response is key in the course of the disease: immunodeficiency states can facilitate a more aggressive course with progression to severe COVID-19 characterized by a deregulated immune response resulting in the so-called “cytokine storm” (10) and complement-induced coagulopathy (2). Moreover, accumulating data are pointing out that COVID-19 is not just a respiratory disease, but a multi-organ dysfunction (11), in which a “bradykinin storm” starting in the lungs may have a pivotal role (12).

Regarding the digestive system, it is noteworthy that both adult and pediatric COVID-19 patients reported gastrointestinal symptoms including diarrhea, vomiting and abdominal pain during course of the disease. The gut symptoms correlate with markers of liver damage (13). Liver injury in patients with COVID-19 is frequent, although mild in nature, with a hepatocellular rather than cholestatic pattern (14). However, severe COVID-19 is accompanied by higher serum transaminases levels (15). In addition to the respiratory system, the gastrointestinal tract is a major infection site of SARS-CoV-2, as ACE2 is highly expressed in proximal and distal enterocytes (16) and viral nucleocapsid protein has been visualized in the cytoplasm of gastric, duodenal, and rectum glandular epithelial cells from a COVID-19 patient (17). Hepatocytes also express detectable amounts of both ACE2 and TMPRSS2, thus they are susceptible of SARS-CoV-2 infection (18). Furthermore, the liver is constantly expose to foreign antigens entering the bloodstream from the gut and keeps a fine balance between the activation of the immune cells for the detection and clearance of pathogens and tolerance towards non-damaging antigens. Due to this reason, the liver has special immune functions and contains the largest number of permanent macrophages, the well-known Kupffer cells (KC). Among their functions, KC are in charge of the clearance of senescent neutrophils and other activated host cells, thus limiting the potential of these cells to produce inflammatory mediators (19).

**Figure 1:**
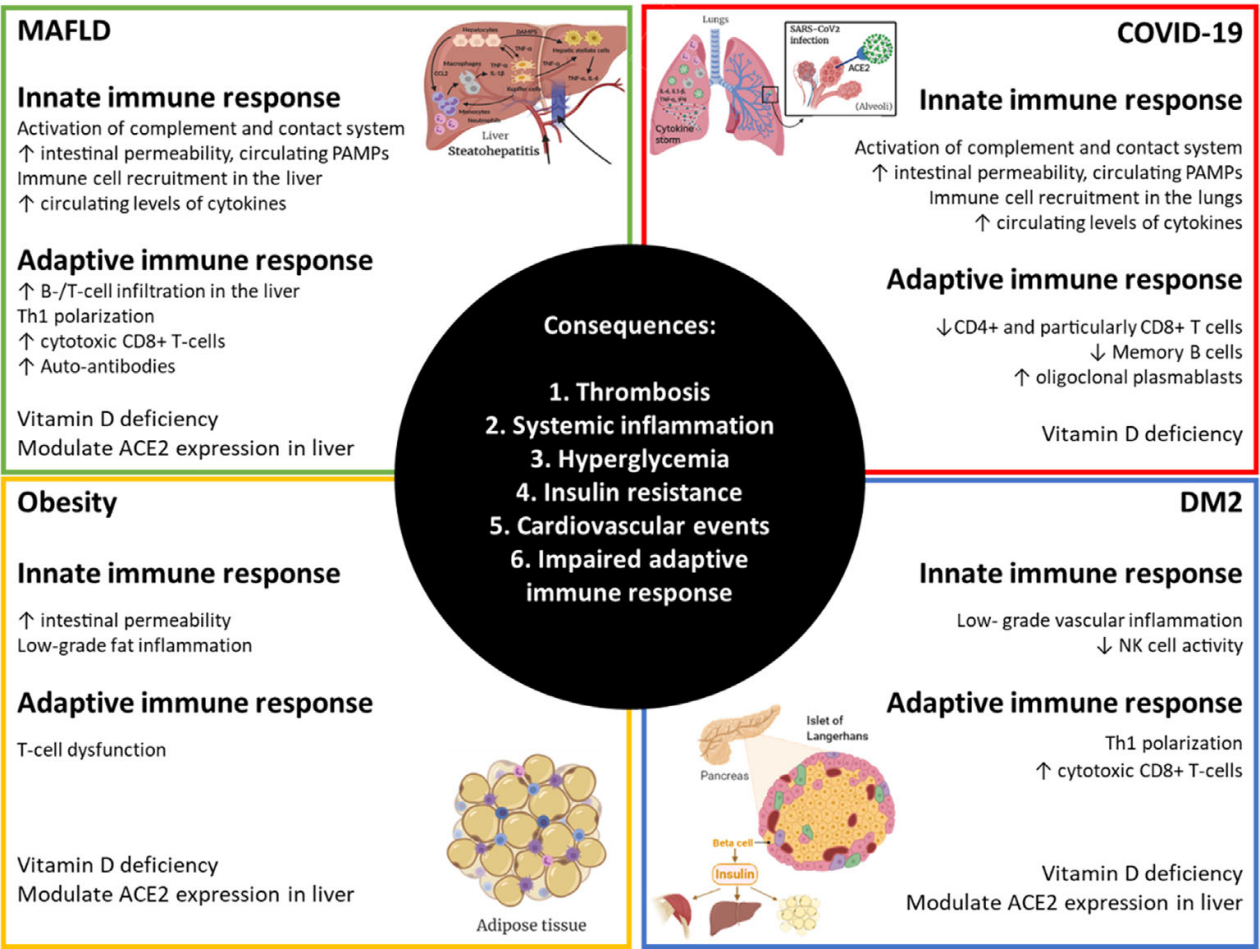
Alterations in immunity that amplify responses to SARS-CoV-2 in MAFLD, obese and DM2 patients. Obesity, diabetes mellitus type 2 (DM2) and metabolic associated fatty liver disease (MAFLD) are very common comorbidities. Each of them has broad effects on the immune system resulting in impairments in normal homeostasis that can lead to more severe COVID-19. Created with BioRender.com.

Older age, male sex, type II diabetes mellitus (DM2) and obesity are mayor risk factors for the development of critically ill COVID-19 (20). Most patients suffering from metabolic associated fatty liver disease - MAFLD, previously known as non-alcoholic fatty liver disease (21) – have all these characteristics (22, 23). Although full description of the mechanisms accounting for DM2 and obesity implication in COVID-19 development have been discussed already (24, 25), **Figure 1** summarizes main contributions of MAFLD, obesity, DM2 and COVID-19 to the overall pathogenesis observed in severe COVID-19 patients. MAFLD includes a spectrum of liver disease defined by an excessive accumulation of fat in hepatocytes, ranging from hepatic steatosis to non-alcoholic steatohepatitis (NASH), liver cirrhosis, and hepatocellular carcinoma (HCC). Whereas steatosis is reversible, later stages cannot be cured and current treatment is limited to lifestyle interventions. Disease progression is characterized by increasing lymphocyte infiltration and inflammation in the liver together with fibrosis and reduced liver function. Recently, new factors contributing to MAFLD have been described, such as gut microbiome alterations, changes in intestinal permeability and bacterial antigen translocation (26, 27). MAFLD prevalence is increasing world-wide together with the pandemic of obesity and metabolic syndrome, affecting already 25% of the adult population globally (23). Moreover, it has been shown that liver fat content is determinant of higher risk of severe COVID-19 in obese patients (28) and the risk of obesity to COVID-19 severity is greater in those with MAFLD than in obese patients without MAFLD (29). Increasing evidence is supporting that MAFLD patients are at risk of developing severe COVID-19 (30–32), even in the absence of its common comorbidities (33) and especially in younger patients (34). Patients with MAFLD had a higher risk of disease progression, higher likelihood of abnormal liver function and longer viral shedding time compared to patients without MAFLD (30). Meta-analysis further confirmed that a high percentage of patients with COVID-19 had MAFLD, and MAFLD increased the risk of disease progression among patients with COVID-19 (35, 36). Liver fibrosis by itself has emerged as a risk factor for severe COVID-19 illness (32, 37) and patients with cirrhosis+COVID-19 had a higher mortality rate compared with those with COVID-19 alone (38, 39). Therefore, we want to focus attention on the high percentage of the population who will suffer from both MAFLD and severe COVID-19 without treatment for any of the diseases and the need to prioritize vaccination of these patients. This review is aimed at summarizing current knowledge about the role of the immune system in the establishment and progression of MAFLD and to discuss its potential implication in the development of severe COVID-19 in MAFLD patients. As a result, we hope to clarify their common pathology, but also uncover new potential therapeutic targets and prognostic biomarkers for further research.

## Alterations of the Innate Immune Response

### Role of Innate Immune Response in MAFLD

There are many components of the innate immune response implicated in the MAFLD pathogenesis and development. Inflammation is an important factor to look out for, due to its capacity to go further and develop HCC, among other tumors (40, 41). Due to the relevant role of innate immunity in MAFLD, in the following sections we will discuss the implication of its different components in MAFLD progression.

#### Complement and Contact Systems

The complement system is activated, among other stimuli, after liver injury, contributing to the development of NASH and HCC (42). Thus, this liver damage reduces the majority of soluble complement proteins levels which lead to an increase of activation markers, and finally, inflammation. Moreover, some complement proteins, like C3 and C4, can act as acute-phase reactants, increasing the systemic inflammation response by 50% (43).

The synthesis of C3 is clearly enhanced in liver pathogenesis (44) and C1q has also been involved in HCC development (45). Other complement proteins that accumulate around hepatocytes with steatosis, such as iC3b, C3d, C4d and C5b-9, are related to some proinflammatory signals such as neutrophils recruitment and cytokine release (46). Therefore, it can be concluded that the inhibition of a permanently activated complement system may ameliorate the liver damage in NASH disease (47).

The contact system is composed of coagulation factors XI (FXI) and XII (FXII), plasma prekallikrein, and high molecular weight kininogen. The final product of the contact system activation cascade is bradykinin, which has vasodilator and pro-inflammatory properties (48). Because of the majority of coagulation factors are formed in the liver and MAFLD has important metabolic implications, a study showed MAFLD patients seem to have increased coagulation factors levels. It suggested a link between the liver fat content and insulin resistance which may be related to FIX and FXI activities. The increase of FXII in murine models could trigger FIX and FXI, which favors thrombosis appearance and propagation in MAFLD (49).

#### Pattern Recognition Receptors (PRR)

The Toll-Like Receptor family (TLR) is an important and well characterized class of cell surface or intracellular PRRs, which are highly expressed in many liver cells under metabolic stress, such as KC, hepatic stellate cells, biliary epithelial cells and sinusoidal endothelial cells (40, 41). Some members of the TLR family (TLR2, TLR4, TLR5, TLR6, TLR7 and TLR9) have been associated to MAFLD pathogenesis. The TLR signal triggers inflammatory pathways in fat tissue and liver, activating transcription factors such as NF-κB and IRFs (Interferon Regulatory Factors) and also several inflammatory cytokines (40, 50, 51). TLR4 is remarkably associated to MAFLD. It binds to the Myeloid Differentiation factor 2 protein (MD-2), and this association confers responsiveness to LPS (52). Actually, both animal models and clinical studies have noticed increased levels of circulating LPS in MAFLD due to the intrinsic endotoxemia caused by factors such as intestinal microbiota, intestinal permeability and high fat and/or sugar diet (HFD) (53–55). This last factor could explain the reason why free fatty acids regulate TLR4 positively in presence of high glucose levels (56). In addition, the TLR4-LPS pathway has also been implicated in MAFLD progression to HCC (57). In HFD, gut microbiota is enriched with gram positive bacteria (58, 59). Taking into account that TLR2 recognizes peptidoglycan, a gram positive bacterial component, its blockade in animal models has also a protective effect in developing insulin resistance, closely related to MAFLD pathogenesis. Actually, some studies have shown that mice with TLR2 deficiency express decreased proinflammatory cytokines by unchaining the inflammasome in KC (50). TLR2 usually forms heterodimers with TLR6, another extracellular receptor increased in NASH patient’s hepatocytes. This heterodimer is also present in lobular inflammation. Therefore, TLR6 has been proposed as a potential biomarker in the development of NASH in MAFLD obese patients (60). TLR7 has also been associated with liver fibrosis (61), although its role in MAFLD is not completely elucidated. According to a study, the presence of TLR7 or its agonists can avoid experimental MAFLD development. An autophagy activation marker seems to be regulated by TLR7 and this process can improve steatosis in MAFLD by stimulating lipid degradation. TLR7 stimulation could be a potential therapeutic target to prevent the consequences of MAFLD, but as it is mentioned before, further investigation is required to clarify TLR7 function in this disease (51). Finally, it should be said that TLR9 is the only intracellular receptor involved in MAFLD pathogenesis. Animal model studies have shown that its activation results in IL-1β production so, along with TLR2 and TLR4, it is strongly involved in NASH and liver fibrosis (50).

The NOD-Like Receptors (NLRs) can form inflammasomes and lead to cell death. NOD-, LRR- and pyrin domain-containing protein 3 (NLRP3) is an intracellular protein complex which plays a key role in the innate immune system triggering several inflammatory components. It has been demonstrated that the lack of NLRP3 inflammasome reduces hepatocyte pyroptosis and, in consequence, inflammation and fibrosis in animal models with NASH. Also, some studies in humans and murine models with liver damage have shown increased levels of NLRP3, caspase 1, IL-1β and IL-18. These facts could play a part in terms of NASH pathogenesis (40, 62).

#### Innate Immune Cells

Signals stimulate several cellular receptors which can activate different cells types in the liver. Around 15% of them are KC (40). After activation, KC can polarize into two phenotipically different forms or also express both at the same time: one more pro-inflammatory (M1) and other mostly characterized by its immunoregulatory properties (M2). Some studies have shown in mice that a HFD benefits the presence of M1 phenotype and the inflammatory response, while the up-regulation of the peroxisome proliferator-activated receptor gamma (PPAR-γ) induces the M1 phenotype polarization to a M2 immunoregulatory phenotype. This fact could prevent the progression of MAFLD disease (63). LPS and other bacterial products activate KC recognition by TLRs and trigger M1 phenotype, which produces several pro-inflammatory cytokines (TNF-α, IL-1β, IL-12), chemokines (CCL2 and CCL5) and damage-associated molecular patterns (DAMPs). These DAMPs promote liver damage through KC activation by TLR pathway, repeating this inflammation process and leading to hepatocyte injury. The accumulation of other products like several free fatty acids, oxidized lipoproteins and other lipids is a very important fact to take into account in experimental MAFLD/NASH pathogenesis because of its involvement in KC activation, causing a bigger response to LPS and therefore, disease progression (40).

Alteration in antigen presenting cells, like dendritic cells (DC), is also relevant in NASH, but its role in this disease is complex and not entirely defined. This could be due to the fact that the two subtypes of conventional DC have opposite roles in terms of NASH activity. A recent study showed that, in patients, cDC2 were positively associated with NASH, and both HLA-DR+CD123-CD11c+CD141+ cDC1 and HLA-DR+CD123+ plasmacytoid DC were inversely correlated with NASH and glucose levels (64).

Natural Killer (NK) cells have a controversial function in NASH pathogenesis: some studies link its activation by different cytokines and ligands to MAFLD/NASH, while others show a reduction in its cytotoxic activity in NASH (65, 66). Additionally, NK T cells (NKT) include two distinct subtypes of cells. Thus, type I NKT is activated by lipid accumulation and may play a pro-inflammatory role in MAFLD, while type II NKT could have an opposite function protecting against liver damage (67, 68). Actually, another study performed on patients showed that the level of liver NKT cells was positively associated with disease stage (69).

These apparently opposite effects of innate immune cells could be due to the plasticity of the immune response and it highlights the need to evaluate changes in phenotype and function longitudinally during liver injury.

#### Cytokines and Other Immune Cells Inflammatory Products

Studies in animal models have shown the involvement of several cytokines in MAFLD, including IL-1β (70), IL-6 (71), TNF-α (71–73) and IFN-α (74). These results are further supported by clinical findings, although some controversy is still present. IL-1β and IL-6 levels were significantly higher in patients with NASH compared with MAFLD and control group, but this study failed in detecting increased levels of TNF-α (75). Serum IL-6 levels were increased in patients with advanced fibrosis compared to patients with mild/no fibrosis (76) and they predict the development of DM2 in women (77). TNF mRNA expression was found increased in hepatic and adipose tissue of NASH patients (78). For instance, TNF-α, among others, featured a clear correlation with transaminase levels and histological severity of MAFLD patients and has been proposed as biomarker of disease progression (79). In contrast, early studies in humans exploring TNF-α blockade as a therapeutic target in metabolic diseases did not show beneficial effects. However, those trials were not well conducted and the clinical designs had some drawbacks such as dosing, duration or presence of confounding factors, among others (80). A recent study has found that levels of circulating pro-inflammatory cytokines were variable in MAFLD patients with or without obesity, but when patients were distributed by the presence of circulating bacterial antigens, a statistically significant increase was observed in serum TNF-α and IL-6 levels in MAFLD patients (26).

Several nuclear transcription factors and some intracellular signaling pathways are involved in MAFLD pathogenesis, but Nuclear factor-kappa B (NF-κB) and c-Jun N-terminal kinase (JNK) are especially remarkable in NASH pro-inflammatory pathways (81, 82). NF-κB is activated by TLRs and triggers the transduction of IL-1β, IL-2, IL-6 and TNF-α (83). JNK overactivation is highly involved in the development of MAFLD and the subsequent liver damage (84). Furthermore, the activation of these pathways links MAFLD and extra-hepatic comorbidities such as insulin resistance and cardiovascular disease (85, 86).

### Innate Immunity and COVID-19 Severity in MAFLD Patients

At this point in the research on COVID-19, it is clear that dysregulated and excessive innate immune responses towards SARS-CoV-2 cause immune damage to the human body. Siddiqi et al. elegantly defined COVID-19 pathogenesis in three phases: early infection, pulmonary phase, and hyperinflammation phase (87). The third and most severe phase is defined by a cytokine storm, which results from a sudden acute increase in circulating levels of different pro-inflammatory cytokines and other related proteins including IL-1β, IL-7, IL-8, IL-9, IL-10, FGF, G-CSF, GM-CSF, IFN-γ, IP-10, MCP-1, MIP-1A, MIP1-B, PDGF, TNF-α, VEGF, C-reactive protein, ferritin and D-dimer (87, 88). MAFLD is associated with chronic, low-grade inflammation in the liver that causes systemic effects, detectable by alterations in circulating immune cells and humoral factors (85). Thus, this precondition would ease the progression of COVID-19 into its severe manifestations, as described hereafter.

#### Complement and Contact Systems

As we already mentioned, the complement system is crucial to trigger an innate immune response to several microorganisms (89) and thus to COVID-19. Actually, some soluble complement proteins, such as C3a and C5a, have proinflammatory functions and they are in charge of immune cell recruitment which can contribute to lung damage in COVID-19 pathogenesis. Although the complement action is not entirely clear about its protection or pathogenicity, a recent study proved the efficacy of a monoclonal antibody against C5/C5a, called eculizumab, in order to ameliorate the pulmonary dysfunction due to COVID-19 (90). Additionally, it has been noticed increased levels of C5a in bronchioalveolar fluid of COVID-19 patients with the most severe symptoms, pointing out the role of this protein in the enhanced inflammation developed in severe COVID-19 patients. Taking this into account, and due to the fact that C5a receptor 1 (C5aR1) levels are also increased in lung neutrophils of severe COVID-19 patients, a study showed anti-C5aR1 therapeutic monoclonal antibodies can fight against C5a action and avoid the infiltration of human myeloid cells in damaged organs, preventing the inflammation developed in severe COVID-19 patients (91). Another study also proved that C3 knockout mice showed lower levels of inflammatory immune innate cells, such as neutrophils and monocytes, leading to reduced SARS-CoV pulmonary injury and suggesting that this virus may activate the complement system (92). As mentioned before, liver injury induces complement activation markers in MAFLD, and due to the fact that this liver damage pattern is also present in COVID-19 (93), MAFLD patients coexisting with COVID-19 infection could have an increased complement function exacerbating the inflammation. The contact system is also an important part of the innate immune defense against viruses. Actually, it has been shown that SARS-CoV-2 penetrates the cell *via* ACE2. Thus, it has been suggested a target therapy consisting of ACE2 direct activation, which avoid SARS-CoV-2 protein S binding to ACE2 (94–96). Moreover, ACE2 has the capacity of degrading des-Arg bradykinin, which is the ligand of the bradykinin receptor type 1 (B1). Once activated, B1 signaling leads to pulmonary angioedema. SARS-CoV-2 infection impairs ACE2 function; therefore, des-Arg bradykinin is accumulated, producing pulmonary angioedema in COVID-19 patients (97). B1 blocking is also a potential strategy to ameliorate pulmonary complications in COVID-19. The complement system, as well as the contact system, seems to be important player in the progression of COVID-19 and, as we mentioned before, their function is altered in MAFLD patients. Although eculizumab is the only approved treatment for humans that inhibits complement cascade at the moment (96), anti-C5aR1 monoclonal antibodies blocking this receptor are a promising and more specific treatment in severe COVID-19 patients due to the fact that C5a is a chemoattractant factor that facilitates the adherence of leukocytes to the endothelium (91).

#### Pattern Recognition Receptors (PRR)

One of the first mechanisms linking COVID-19 severity and MAFLD affects the most basic elements of innate immunity: physical barriers. Several authors suggest that the increased risk observed in MAFLD patients is driven by SARS-CoV-2 infection of the gut, which exacerbates an existing state of intestinal permeability and mucosal inflammation, increasing the transmission of pathogen-associated molecular patterns to the liver and affecting systemic immune response (98–101). Supporting data is preliminary, but some authors have already shown that COVID-19 patients have altered fecal microbiota (102–105) and plasma markers of gut leakage and inflammasome activation are increased in COVID-19 patients, especially in those with cardiac involvement (106).

Some evidence also pointed out at TLR signaling as possible impaired mechanisms in severe COVID-19. On one hand, bioinformatics studies have shown that SARS-CoV-2 genome possess multiple single-stranded RNA sequences probably recognized by TLR7 and 8 (107). A case-series article identified four young men from two families carrying rare and inactivating mutations in TLR7 who suffered from severe COVID-19 in the absence of common comorbitidies (108). Although these mutations are unlikely to be an explanation for severe COVID-19 in the general population, other mechanisms accounting for TLR7 signaling impairment could result in a similar situation. Accordingly, some authors hypothesize that chronic stimulation of TLR7 by intrinsic substrates could lead to a desensitization of TLR7 signalling. Therefore, the immune response in those patients will be delayed upon viral infection, but when resensitization finally occurs, it leads to an overwhelming TLR7 response (109). As mentioned before, TLR7 seems to perform a protective role in liver injury (61) and MAFLD (51), but it has been also described that liver injury can promote pulmonary inflammation through the activation of TLR7/8 in alveolar macrophages. Based in human samples and animal models, authors found that injured hepatocytes release miRNA-122, which is preferentially transported to the lungs where it triggers TLR7/8 signaling eliciting macrophage inflammatory responses (110). On the other hand, TLR3 and TLR4 knock-out mice have shown its relevance in preventing SARS-CoV lethal infection, in which TRIF adaptor protein has a central role (111). In this sense, a small study analyzed PBMCs transcriptomics in patients with COVID-19 and observed that the expression of TLR4 and downstream signaling molecules were significantly upregulated. These authors also showed that SARS-CoV-2 proteins increased the expression of the TLR4 ligand S100A8/A9 in PBMCs *in vitro*, proposing that SARS-CoV-2 infection can product a feed-forward loop through TLR4 activation that sustained the inflammation in COVID-19 patients (112). As mention before, MAFLD patients have increased intestinal permeability, resulting in increased circulating levels of LPS (113). In conclusion, patients suffering from MAFLD could suffer a chronic stimulation of TLR7/8 and TLR4 in the alveolar macrophages, which make them more vulnerable to severe COVID-19.

Finally, SARS-CoV-2 is able to induce NRPL3 inflammasome (114–116), which is also a major contributor to hepatocyte death in NASH. Recently, it has been shown that the magnitude and course of NRPL3 activation has an important role in the clinical outcome of COVID-19 patients during their hospitalization period. NRPL3 presence in PBMCs of these patients, together with high levels of casp1p20, among others, support this statement and suggest inflammasome can act not only as a marker of severity, but also prognosis and even as a therapeutic target (117).

#### Innate Immune Cells

The innate immune system appears to have an opposite role to adaptive responses in SARS-COV2 infections; several innate immune cells sometimes contribute to the progression of the disease (118). Actually, lung inflammation caused by COVID-19 can be aggravated because of macrophage activation syndrome and its production of several inflammatory cytokines (IL-6, IL-7, TNF), chemokines (CCL2, CCL3, CXCL10) and the soluble form of α-chain of the IL-2 receptor (119, 120). In COVID-19 patients, hepatocellular liver damage is apparently frequent, and knowing that liver macrophages are able to produce a great variety of cytokines, it could be suggested a leaning towards M1 macrophages polarization of these immune cells thus contributing to the development of COVID-19 in MAFLD (93). Not only macrophages take part in COVID-19 pathogenesis, other immune innate cells such as monocytes and neutrophils are involved, and their levels in COVID-19 patients were found increased (121). Severe COVID-19 patients showed high levels of two essential monocyte recruitment chemokines: CCL2 and CCL7 (122). In another study of COVID-19 patients who needed ICU hospitalization, a remarkable CD14+CD16+ monocyte levels that also produce IL-6 were noticed (120). The inflammatory environment induced by these cells cause an excessive inflammation which could be even more dangerous than the virus infection itself (121). Additionally, the sex differences in COVID-19 patients are an interesting fact because they could be related to hormonal dependency: some immune innate cells such as neutrophils, macrophages, DC and NKs depend on estrogen and testosterone to mature and differentiate, without forgetting they can be modulated by individual variability and genetic background (123). Interestingly, a recent published study reported that MAFLD could also have sex specific preferences: several metabolic pathways and other inflammatory processes in the liver are regulated by estrogen receptors (124).

#### Cytokines and Other Immune Cells Inflammatory Products

As already mentioned, cytokines emerged as key players in severe COVID-19, correlating directly with lung injury and multiorgan failure. Actually, serum IL-6 level has been widely accepted as prognostic marker in COVID-19 as its elevation is the most frequently reported (88). For this reason, tocilizumab, a humanized anti-IL-6-receptor monoclonal antibody, has been proposed as a treatment for severe COVID-19 and used as an emergency and compassionate treatment (125, 126). It is currently under evaluation in clinical trials, with promising preliminary results (127, 128). Several clinical trials are also evaluating the efficacy of IL-1β inhibitors, such as anakinra and canakinumab, in preventing COVID-19 pneumonia and its associated cytokine storm; but, for now, the only evidence came from CAN-COVID interim analysis, in which canakinumab has failed to meet primary endpoints (129). Nonetheless, the cytokine storm involves lots of different pro-inflammatory cytokines; therefore, general immunosuppression could be even more effective. At this regard, corticosteroids have also been evaluated for the treatment of severe COVID-19 and, although earlier meta-analysis conclude with negative results (130), CoDEX clinical trial showed increased ventilator-free days in treated patients (131) and the WHO REACT Working Group further support lower 28-day all-cause mortality in critically ill COVID-19 patients receiving systemic corticosteroids (132). An important issue in the context of cytokine inhibition is to determine the right timing for treatments. For instance, IFN has been recommended as first-line antiviral in SARS-CoV-2 infection, although evidence is weak for now (133). Taking into consideration that MAFLD patients have increased basal levels of circulating cytokines, these patients may benefit for early intervention with immunosuppressive therapy. However, liver functions may be compromised in MAFLD patients and, accordingly, any therapeutic intervention should consider that drug metabolism could be impaired and further result in liver injury (134, 135). On the other hand, as mentioned previously, the immune response is dynamic and it is possible that many of the failed anti-cytokine therapies in COVID-19 may relate to the time of introduction of the treatment in the course of the disease.

**Figure 2:**
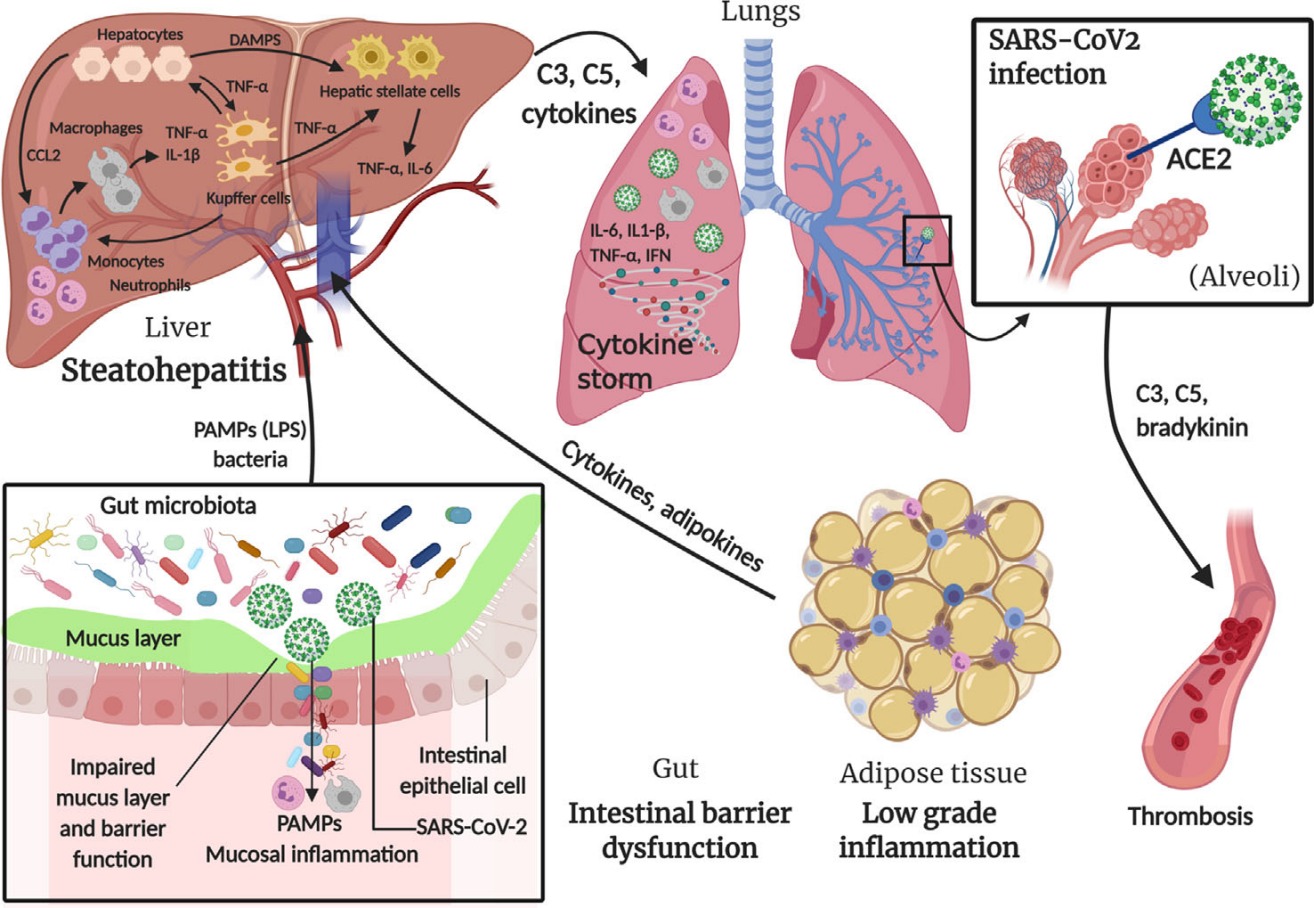
Involvement of innate immune system in MAFLD and SARS-CoV-2 infection. In metabolic associated fatty liver disease (MAFLD), intestinal barrier dysfunction caused by intrinsic endotoxemia due to alterations in gut microbiota, adipose tissue low grade inflammation and the excess of fat in hepatocytes, lead to a series of events triggering the innate immune response in order to restore the perturbed environment. SARS-CoV-2 infection also contributes to intestinal mucosal inflammation, in addition to main lung inflammation caused by the virus entry through angiotensin-converting enzyme 2 (ACE2) in alveoli, which can also lead to thrombosis through a “bradykinin storm”. All these triggers lead to a massive “cytokine storm” with multiorgan effect. Created with BioRender.com.

**Figure 2** summarizes the main concepts discussed in this section.

## Alterations of the Adaptive Immune Response

### Role of Adaptive Immune Responses and Innate-Adaptive Interplay in MAFLD

Although the implication of innate immune response and inflammation in MAFLD progression seems clear, recent findings have also uncovered a role of adaptive immune responses. As stated before, lipid accumulation in hepatocytes is an early finding in MAFLD and ROS generation is considered one of the main drivers of the diseases. In this sense, oxidized phospholipids and reactive aldehydes generated during lipid peroxidation form antigenic adducts with cellular macromolecules known as oxidative stress derived epitopes (OSEs) (136). These epitopes trigger both humoral and cellular adaptive immune responses. Much of the role of adaptive immunity in MAFLD is supported by the results obtained from different experimental models, including obesity related MAFLD (mice under different types of HFD) and NASH development in lean individuals (such as mice in methionine and choline deficient diet or similar diets, MCD). In this sense, comparison between mice living in specific pathogen-free (SPF) conditions with those housed on non-SPF showed that the latter had higher memory and effector T cells both under normal and HFD. These results were also observed in the liver: more than 95 and 85% of CD4+ and CD8+ T cells, respectively, expressed the effector memory T cell phenotype in non-SPF mice on HFD, which was accompanied by severe steatosis, lobular inflammation, hepatocellular ballooning and destroyed lobule structure while only some SPF mice displayed a mild fat accumulation in the liver (137). On the other hand, mice following a HFD showed reduced antigen-specific humoral and cellular immune responses after receiving hepatitis B vaccine due to diminish antigen processing and presentation (138). Recently, it has been also reported that follicular T helper cells are impaired in patients with advanced liver cirrhosis due to increased IL-2 signaling (139). These results give an early notion that adaptive immunity activation directly impact on MAFLD severity and, at the same time, MAFLD and cirrhosis result in impaired adaptive immunity activation towards other antigens. In this sense, exploring the immune response to SARS-CoV-2 vaccination seems mandatory in MAFLD and comparison between obese and non-obese patients.

Elevated titers of IgG anti-OSEs such as malondialdehyde adduct with human serum albumin (MDA-HSA), arachidonic acid hydroperoxide adduct with human serum albumin (AAHP-HSA) and oxidized cardiolipin (Ox-CL) have been found in MAFLD patients compared with controls. Moreover, 29–39% of total MAFLD patients had anti-OSEs IgG titers above the 97.5^th^ percentile in controls, which defines a positive titer. MAFLD patients with positive anti-MDA-HSA antibodies had a threefold higher risk of having advanced fibrosis or cirrhosis compared with patients whose antibody titers were within the control range (140). Similar results were observed in pediatric MAFLD patients; in this case, authors found that 63% of patients had circulating IgG against anti-MDA-HSA above the control threshold. At the histology, patients with elevated anti-MDA-HSA antibodies showed higher scores of lobular inflammation than subjects with antibodies within control range (141). In an independent and more recent study, humoral immunity against OSE was confirmed by measuring circulating IgG anti-MDA adducts. They found that 43% of MAFLD/NASH patients had titers of anti-OSE IgG above the control threshold. The prevalence of advanced fibrosis or cirrhosis was higher among the subjects with elevated anti-OSE IgG (142). In this line, studies with MCD fed mice showed that the extension of liver injury and lobular inflammation paralleled the development of anti-MDA IgG antibodies and CD4+ and CD8+ T-lymphocytes responsive to the same antigens. Besides, further treatment with MDA-adducted bovine serum albumin stimulated transaminase release, lobular inflammation, and the hepatic expression of proinflammatory cytokines in MCD-fed mice, involving liver recruitment of the Th1 cells (143). These studies point out the early implication of humoral immunity in the recruitment of immune cells to the liver in MAFLD and the sustained autoimmune response as a factor contributing to disease progression. On the contrary, in a smaller cohort, Hendrikx et al. described no differences in plasma IgG anti-OSEs titers in MAFLD patients and healthy controls. Besides, they found decreased IgM anti-OSEs titers in MAFLD patients, although only IgM titers towards the specific malondialdehydeacetaldehyde P1 mimotope remained significant after adjusting for total IgM levels. They also showed that IgM titers against P1 mimotope inversely correlate with markers of obesity, systemic inflammation and liver damage, gaining a protective role (144). Moreover, low anti-adipocyte IgG antibodies have been observed in MAFLD patients in comparison to controls, whereas anti-adipocyte IgM antibodies were increased and correlated with portal inflammation (145). Finally, autoimmune hepatitis and common autoantibodies are more prevalent in MAFLD patients than in the general population (146–149), although its relevance in disease progression is unclear (149, 150). Thus, humoral response in human MAFLD is still controversial and deserves further research.

Cellular adaptive responses have been also described in MAFLD patients. 63% of MAFLD/NASH patients showed CD20+ B-cell and CD3+ T-cell aggregates in liver biopsies as determined by immunostaining (151). B and T lymphocyte infiltration in the liver of experimental models of MAFLD has been widely described (152–157). Patients with high B-/T-cell infiltration had elevated anti-OSE IgG titers as well as higher scores of lobular inflammation and fibrosis than the subjects with low/mild infiltration. The number and size of lymphocyte aggregates positively correlated with circulating IFN-γ levels, lobular inflammation score and fibrosis staging (142). NASH patients showed increased serum B-cell Activating Factor (BAFF) (158). Although little is known about the role of B lymphocytes in MAFLD pathogenesis, in mice receiving the MCD diet, hepatic B2-lymphocytes significantly declined in parallel with the onset of steatohepatitis and increasing titers of circulating anti-OSE IgG, accompanied by increased markers of lymphocyte activation and plasmablasts and cells expansion. Selective depletion of B2-lymphocytes resulted in reduced liver plasma cell maturation, anti-OSE IgG production, impaired liver recruitment of T lymphocytes, ameliorated lobular inflammation score and the prevalence of necrotic foci without affecting the extension of steatosis upon the induction of NASH. B-cell responses in NASH were associated to the up-regulation in the liver of BAFF. Thus, BAFF neutralization also ameliorated histological scores for steatosis and lobular inflammation as well as ALT release, liver triglycerides and hepatic expression of pro-inflammatory mediators (142). Moreover, B lymphocytes seemed to infiltrate earlier in the liver of HFD mice than T cells (159).

On the contrary, T cell alterations both in peripheral blood and intrahepatic infiltrates from MAFLD patients have been widely described. In this sense, several lines of evidence suggest that NASH should be considered a Th1-polarized disease (64, 74, 160–164). Circulating CD4+ cells rose in adult NASH patients (160, 162), together with an increased proportion of memory CD45RO+ cells and decreased of naïve CD45RA+ (151, 160). Similarly, CD8+CD45RO+ subpopulation was increased while CD8+CD45RA+ declined (151, 160). In pediatric NASH, whereas CD45RO and CD45RA subpopulations were similarly distributed among circulating CD4+, CD8+CD45RO and CD8+CD45RA subpopulations were found simultaneously increased in patients, which refers to a greater pool of CD8 T cells undergoing activation (161). Moreover, a higher frequency of IFN-γ-producing Th1 lymphocytes was observed as well as CD8+ cells retaining a cytotoxic phenotype (160, 161). In liver biopsies, IFN-γ-producing CD8+ cells were also increased both in adult and pediatric NASH (64, 160, 161). CD8+ T lymphocytes localized within inflammatory foci in close proximity to steatotic and ballooned hepatocytes and showed associations with lobular inflammation, ballooning and hepatic genes related to cytotoxic and IFN-γ responses, T helper differentiation and TNF-α signaling, strongly suggesting the presence of a local cytotoxic response in the liver (64). Other authors have observed that also the frequency of IL-4+ Th2 cells among CD4+ T cells was significantly elevated in patients with MAFLD and NASH in comparison with controls (151, 162). Moreover, analysis of intrahepatic lymphocytes showed significantly higher frequencies of intrahepatic IL-17, IL-4, and IFN-γ-producing T cells compared with peripheral blood. The greatest difference between intrahepatic and peripheral T cells was seen for the frequency of IFN-γ + cells among CD4+ T cells both for MAFLD and NASH. In livers from MAFLD and NASH groups, up to 44% of CD4+ T cells expressed the activation marker HLA-DR, thus contributing actively to pathogenesis *in situ*. However, Th17 cells were more frequent in the liver of patients with NASH in comparison with hepatic tissue from patients with MAFLD, which could differentiate disease stage (162). Regarding regulatory T-cells (Tregs), MAFLD patients showed a significantly lower frequency of naïve Tregs (CD4+CD45RA+CD25++) among CD4+ T cells in peripheral blood in comparison with controls, while NASH patients had an even lower frequency of these cells. The opposite was true for activated Tregs (CD4+CD45RA-CD25+++), suggesting increased turnover/consumption of Tregs in patients as a result of increased activation of the naïve Tregs (162). Another study reported that Foxp3+ Tregs are increased in the liver of NASH patients and its frequency among T cells correlated to higher NASscore (165). Moreover, oral treatment with anti-CD3 monoclonal antibodies improved serum transaminases and fasting plasma glucose in a small cohort of NASH patients through increasing circulatory Tregs (166). Although evidence is still scarce, we can hypothesize that Tregs may get activated and infiltrate to the liver in MAFLD patients as a compensatory mechanism for the enhanced local immune response, which in the context of sustained liver damage could favor the development of liver cancer (167). On the contrary, studies in the liver of HFD fed mice have shown that percentages of CD4+IFN-γ+ Th1 cells and CD4+IL-17+ Th17 cells were increased remarkably, while CD4+CD25+Foxp3+ Treg cells were decreased significantly (168, 169), which parallels the imbalance in T cell activation in the mesenteric lymph node. Chemotaxis of CD4+ from mesenteric lymph nodes to the liver was demonstrated, thus linking gut immunity alterations and MAFLD (168, 170). In this sense, although lymphocyte infiltration in the liver is mainly associated with bad prognosis in MAFLD, several studies in experimental models have shown that CD4+ T cell depletion could increase the risk of HCC development (171–173). On the contrary, maintained CD8+ cytotoxic responses seem to favor carcinogenesis (156, 157).

On the other hand, conflicting results have been reported. For instance, no differences between control, MAFLD and NASH in B cell or T cell populations have been described (174). Other studies have shown a reduction of circulating CD8+ T cells in MAFLD patients in comparison to control healthy subjects (151) or between steatosis stages (175). Moreover, circulating Th2 cells were observed increased while Th1 cells, Th17 cells and Tregs cells had similar frequencies in MAFLD patients compared to healthy controls (151). These contradictory results could be due to the common comorbidities associated to MAFLD, namely DM2 and obesity, and its different representation in the cohorts. Thus, several authors have analyzed the different immune response in MAFLD, DM2 and obese patients. Regarding the humoral response, MAFLD patients diagnosed with diabetes or hyperlipidemia were found to have significantly lower levels of anti-adipocytes IgG antibodies when compared with MAFLD patients with none of the comorbidities. Furthermore, anti-adipocytes IgM correlated positively and significantly with body-mass index while the contrary was true for anti-adipocytes IgG (145). After multiplex determination of cytokines in obese patients, Vonghia et al. concluded that DM2 patients showed a disturbed Th1/Th2 balance towards Th1 polarization, but, at the intrahepatic level, a mixed Th1 and Th2 impairment occurred and Th2 response was common to DM2 and NASH. Moreover, patients with advanced fibrosis showed higher intrahepatic INF-γ and IL-1β, which can stimulate the cells towards a pro-inflammatory Th1 phenotype (176). Besides, other authors stated that the hepatic expression levels of several mediators of the immune response are modified in all morbidly obese patients, regardless steatosis or inflammation, while NASH appears preferentially associated with a better antigen presentation and a Th1 response, highlighting again the relevance of adaptive immunity in MAFLD progression (163). Finally, authors analyzed the association of differentially expressed genes and immune cell populations in a large cohort of obese patients showing common metabolic comorbidities. IL-10+ CD4 T lymphocytes and cytotoxic CD8 T were positively associated with lobular inflammation, ballooning and glucose levels, thus linking NASH activity and DM2. Th2 lymphocytes and Tregs were mostly negatively associated with NASH and glucose parameters (64).

### Adaptive Immunity and COVID-19 Severity in MAFLD Patients

**Figure 3:**
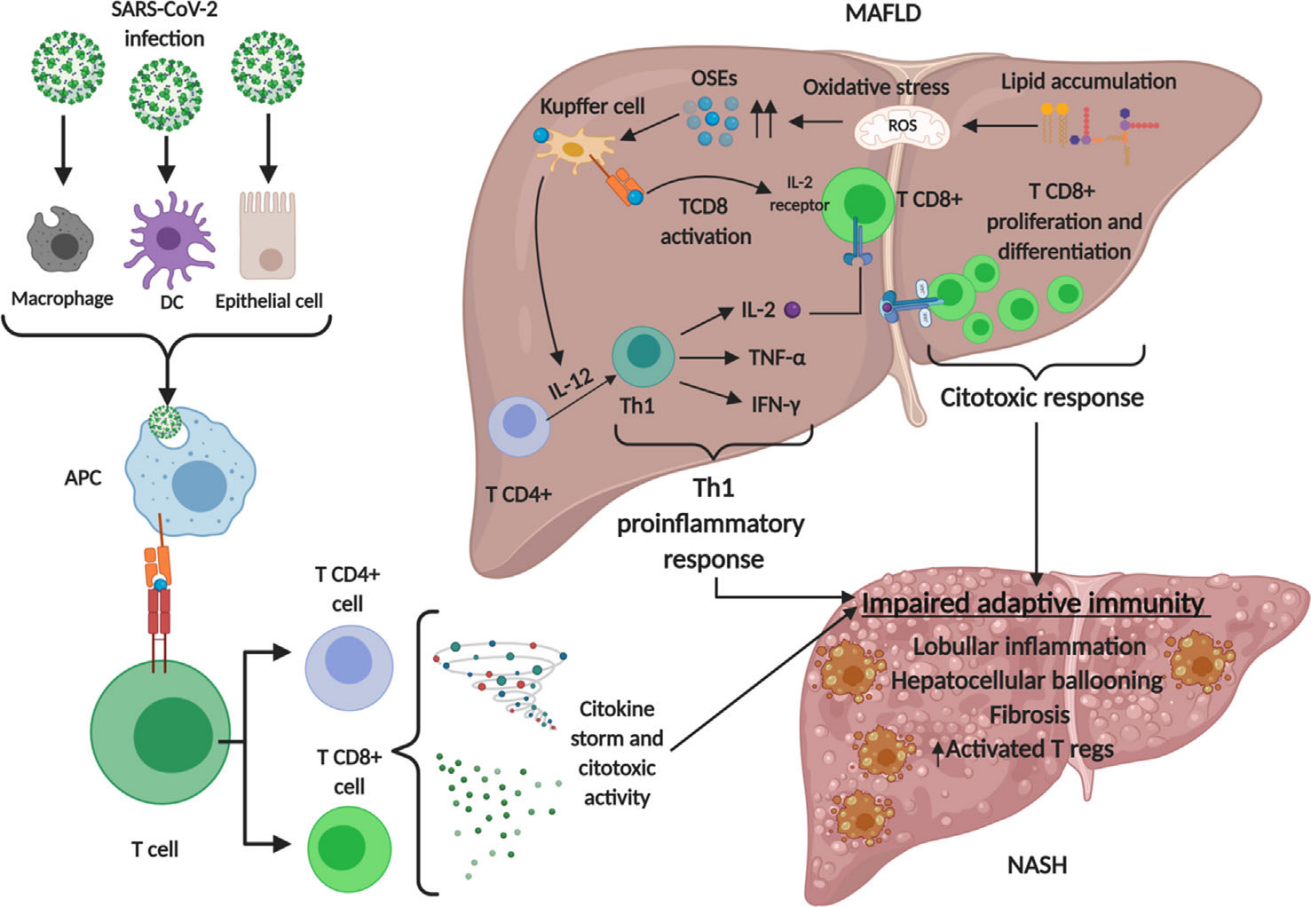
Involvement of adaptive immune system in MAFLD and SARS-CoV-2 infection. Lipid accumulation in metabolic associated fatty liver disease (MAFLD) causes oxidative stress and lipid peroxidation, generating oxidative stress derived epitopes (OSEs). These products lead to adaptive immune stimulating T cells toward Th1 proinflammatory phenotype and activating T CD8 cells which express interleukin 2 (IL-2) receptor to develop a cytotoxic response. These events, together with SARS-CoV-2 infection lead to an impaired adaptive immune response. Regulatory T cells (Tregs) infiltrate to the liver as a compensatory mechanism for the enhanced local immune response. Created with BioRender.com.

Lymphopenia is a common feature of severe COVID-19, characterized by drastically reduced absolute numbers of CD4+ and particularly CD8+ T cells which correlates with COVID-19 severity and associated mortality (177, 178). Levels of T cell surface molecules (CD4, CD8 and CD2), T cell migration stimulators (DDP4), TCR signaling kinases (ZAP70, LCK and FYN) and MHC class II molecules (HLA-DRA, HLA-DRB1, HLA-DRB4 and HLA-DRB5) were also significantly lower in patients with severe disease (179). Peripheral CD8+ T cells from patients with COVID-19 express high levels of exhaustion markers, including programmed cell death protein 1 (PD1) and T cell immunoglobulin mucin-3 (TIM3); of note, this expression pattern was more pronounced among patients who required intensive care than in patients with mild disease (180). Analyses of circulating B cells showed expansion of oligoclonal plasmablasts and reduced memory B cell frequencies in patients with severe COVID-19 compared with responses in patients with mild disease or healthy individuals (181, 182). Taking all together, severe COVID-19 patients show a global impairment in the adaptive immune response. As stated before, adaptive immune responses towards auto-antigens in NASH result in lymphocyte infiltration in the liver, with a pro-inflammatory Th1 phenotype and increased consumption of Tregs. These mean a basal increased exhaustion of the adaptive immune system in NASH patients in comparison with healthy people, which could lead to a worse adaptive response towards SARS-CoV-2 infection. Th1 phenotype is also observed in lungs during SARS-CoV-2 infection, as macrophage phagocytosis of infected alveolar epithelial cells stimulate T cells to secrete IFN-γ and favors alveoli inflammation, forming a detrimental positive loop for severe COVID-19 (134). Moreover, a preliminary study with a Chinese cohort of COVID-19 patients showed that patients with MAFLD and increased neutrophil-to-lymphocyte ratio on admission are at substantially higher risk of severe COVID-19, irrespective of age, sex and metabolic comorbidities, indicating that the inability to transit from innate immune responses to adaptive ones is determinant in the progression of COVID-19 in MAFLD patients (183). Nonetheless, evidence is still very scarce and mechanisms underlying the pathophysiological links between metabolic syndrome and COVID-19 are mainly unproven. **Figure 3** summarizes the main concepts discussed in this section.

## Other Factors Involved in MAFLD and COVID-19 Pathophysiology

Other factors could be responsible for the high incidence of severe COVID-19 in MAFLD patients. For instance, ACE2 is normally expressed in low amounts in cholangiocytes and hepatocytes, but it is increased in chronic liver damage and in experimental set-ups of diet-induced MAFLD, where it may exert anti-obesity and anti-inflammatory effects. Therefore, liver injury could lead to increased viral load and worsened effects of COVID-19 (184). In this line of research, SARS-CoV-2 entry factors are differently affected by DM2 and MAFLD in the liver of obese patients. While obese women with DM2 have lower expression of ACE2 and TMPRSS2 than obese normoglycemic women, obese men with NASH show markedly higher levels of these genes (18), which may explain the higher risk of severe COVID-19 in these patients (29). Very preliminary data suggest that liver injury in COVID-19 is more likely due to the exacerbated immune responses than for direct viral infection, but, to affirm this, postmortem liver biopsy was only performed in one patient (30).

Furthermore, it has been shown that COVID-19 severe patients also have downregulation of some classes of metabolites as well as dysfunctional metabolic processes, leading to a loss of important circulating nutrients. This fact, together with high cytokine levels and a proinflammatory environment, contribute to a possible hepatic dysfunction worsening the patient condition (185).

As mentioned before, the liver has special immune characteristics. Besides, its role as a secretory organ, particularly with respect the regulation of coagulation and hemostasis, makes it indispensable for intertissue communication. The steatotic and injured liver could therefore produce hepatokines that may alter the function of other systems, making MAFLD a multi-systemic disease. These molecules have been implicated in the development of increased adipocity, kidney injury, DM2 and cardiovascular disease (186). All these comorbidities are risk factors for severe COVID-19, and therefore evaluating the role of hepatokines in the progression of COVID-19 would be reasonable, but no attempts have been performed until day.

## Conclusion

Increasing evidence is confirming an enhanced risk of severe COVID-19 in MAFLD patients, together with other common comorbidities of this disease such as DM2 and obesity. In this sense, a well-established paradigm in MAFLD pathogenesis is the chronic low-grade inflammation, which is the perfect niche for the development of a cytokine storm upon SARS-CoV-2 infection. As discussed along the text, TLR signaling might be sustained in MAFLD and COVID-19, leading to a hyperactivation of neutrophils and macrophages that produce large systemic levels of proinflammatory molecules. Not only regarding cytokines, the dysfunction of the innate immune response is key in both diseases, affecting the integrity of physical barriers, especially in the intestine; and the complement and contact systems, which are also responsible for the severe and long-lasting manifestations of COVID-19. Furthermore, the needed transition between innate and adaptive immune responses seems to be impaired in severe COVID-19. The dysregulation of adaptive responses is already present in MAFLD patients, who also have a proinflammatory T cell response and exhaustion of Tregs. Besides, humoral responses are activated towards auto-antigens in some cases. In this setting, proper adaptive immunity could not be expected.

Taking into consideration all these pathological mechanisms, several therapeutic approaches have been proposed. ACE2 direct activation has been considered to prevent SARS-CoV2 infection. Additionally, the efficacy of anakinra and canakinumab (IL-1β inhibitors) in preventing COVID-19 pneumonia and its associated cytokine storm is also being tested in several clinical trials; but, for now, canakinumab has failed to meet primary endpoints. To keep combating the inflammation, eculizumab is at the moment the only medication approved for humans to prevent the complement cascade in order to ameliorate the pulmonary dysfunction due to COVID-19, but also specific C5aR1 blockade is a very promising therapy to fight against severe COVID-19. General immunosuppression, through inhibition of NF-κB or treatment with corticoids reduces the cytokine storm, helping to diminish the inflammatory environment created by both MAFLD and COVID-19 diseases.

Last of all, immune independent mechanisms can also account for the increased risk of severe COVID-19 in MAFLD patients, but current studies are very limited.

## Author Contributions

Conceptualization, JC and ML-H. Writing—original draft preparation, PL and MA-P. Writing—review and editing, DS, MA-L, JC, and ML-H. All authors contributed to the article and approved the submitted version.

## Funding

This research received funding by the ISCIII (COV20/0170 and PI19/01509) and Cantabria Goverment (2020 UIC22-PUB-0019) to ML-H.

## Conflict of Interest

The authors declare that the research was conducted in the absence of any commercial or financial relationships that could be construed as a potential conflict of interest.
